# PACAP38/PAC1 Signaling Induces Bone Marrow-Derived Cells Homing to Ischemic Brain

**DOI:** 10.1002/stem.1915

**Published:** 2015-03-24

**Authors:** Chen-Huan Lin, Lian Chiu, Hsu-Tung Lee, Chun-Wei Chiang, Shih-Ping Liu, Yung-Hsiang Hsu, Shinn-Zong Lin, Chung Y Hsu, Chia-Hung Hsieh, Woei-Cherng Shyu

**Affiliations:** a Center for Neuropsychiatry and Translational Medicine Research Center, China Medical University and HospitalTaichung, Taiwan; b Department of Nursing, College of Medicine and Nursing, Hungkuang UniversityTaichung, Taiwan; c Department of Neurosurgery, Taichung Veterans General HospitalTaichung, Taiwan; d Graduate Institute of Medical Sciences, National Defense Medical CenterTaipei, Taiwan; e Graduate Institute of Basic Science, China Medical UniversityTaichung, Taiwan; g Graduate Institute of Immunology, China Medical UniversityTaichung, Taiwan; h Graduate Institute of Clinical Medical Science, China Medical UniversityTaichung, Taiwan; f Department of Pathology, Buddhist Tzu-Chi General Hospital, Tzu-Chi UniversityHualien, Taiwan; i Department of Biomedical Informatics, Asia UniversityTaichung, Taiwan

**Keywords:** Bone marrow-derived cells, PACAP38/PAC1 signaling, Hypoxia-inducible factor-1α, Vascular niche, Stroke

## Abstract

Understanding stem cell homing, which is governed by environmental signals from the surrounding niche, is important for developing effective stem cell-based repair strategies. The molecular mechanism by which the brain under ischemic stress recruits bone marrow-derived cells (BMDCs) to the vascular niche remains poorly characterized. Here we report that hypoxia-inducible factor-1α (HIF-1α) activation upregulates pituitary adenylate cyclase-activating peptide 38 (PACAP38), which in turn activates PACAP type 1 receptor (PAC1) under hypoxia in vitro and cerebral ischemia in vivo. BMDCs homing to endothelial cells in the ischemic brain are mediated by HIF-1α activation of the PACAP38-PAC1 signaling cascade followed by upregulation of cellular prion protein and α6-integrin to enhance the ability of BMDCs to bind laminin in the vascular niche. Exogenous PACAP38 confers a similar effect in facilitating BMDCs homing into the ischemic brain, resulting in reduction of ischemic brain injury. These findings suggest a novel HIF-1α-activated PACAP38-PAC1 signaling process in initiating BMDCs homing into the ischemic brain for reducing brain injury and enhancing functional recovery after ischemic stroke. Stem Cells *2015;33:1153–1172*

## Introduction

Cerebral ischemia creates a specific niche for stem cells in the affected brain region which is enriched with soluble factors including cytokines, chemokines, and growth factors secreted from the adjacent stroma, neurons, and peripheral blood in response to ischemic insult [1,2]. Some of these factors including stromal cell-derived factor 1α (SDF-1α) and vasoactive intestinal peptide (VIP) with angiogenic actions are regulated by the oxygen-sensitive hypoxia-inducible factor-1α (HIF-1α) [3]. Pituitary adenylate cyclase-activating polypeptide (PACAP), belonging to the same neuroendocrine peptide family as VIP, exerts a multitude of actions including neuroprotection and angiogenesis [4] by interacting with VIP/PACAP receptor 1 (VPAC1) and selective PACAP receptor 1 (PAC1) [5]. Although expression of PACAP is also induced by ischemic injury [6] as VIP, whether HIF-1α activation involved in upregulation of PACAP expression following ischemia has not been reported.

We and others have previously reported that the ischemic microenvironment expresses signals that promote homing of BMDCs into ischemic areas [7,8]. The ability of stem cells to relocate and occupy niches is essential for normal stem cell biology. Although some factors that drive homing of BMDCs to vascular niches within bone marrow using several molecules under physiological conditions and following transplantation has been identified [9], how BMDCs home to their niche in the ischemic brain is not well known. For example in SDF-1α/CXC receptor type 4 (CXCR4) signaling system, SDF-1α also plays an important role in trafficking of circulating hematopoietic stem cells (HSCs) by interacting with its receptor of CXCR4 from peripheral circulation to the ischemic brain [7]. Actin polymerization and upregulation of integrins occur in circulating HSCs, resulting in chemotaxis toward the source of SDF-1α [10]. Regarding the impact of PACAP in cell trafficking, previous reports demonstrated that it promoted neural progenitor cells (NPCs) migration and trafficking via activation of PAC1 [11–13]. Accordingly, based on the crucial link that CD34^+^ HSCs preferentially express PAC1 [14,15], we decided to pursue the idea that PACAP38/PAC1 axis plays a critical role involved in BMDCs homing to the vascular niche of ischemic brain.

HIF-1α, binding to the putative hypoxic-responsive element (HRE) of gene promoters is the central transcriptional mediator of the cellular response of hypoxia and ischemia [16–18]. Stem cells trafficking are modulated by the HIF-1α gradient of the vascular niche [19,20]. In this study, we hypothesize that upregulation of PACAP38 expression under hypoxia/ischemia is through HIF-1α binding putative HRE on the PACAP38 promoter. We further assume that PACAP38 actions in reducing hypoxia/ischemia injury and promoting stroke recovery are mediated by its interaction with PAC1, establishing the PACAP38-PAC1 signaling cascade to mobilize BMDCs for homing to the vascular niche in the ischemic brain.

## Materials and Methods

### Immunohistochemical Analysis of Human Brain After Stroke

We investigated autopsy brain specimens from nine cases of fatal ischemic stroke (disease duration ranged from 10 hours to 7 days) treated at the Department of Neurology, China Medical University Hospital. Autopsy surgery was performed within a mean of 6 hours after death (ranging from 4 to 12 hours). Three patients who died of a non-neurological cause served as controls (autopsies were conducted at 0.5 day, 3 days and 5 days). The study protocol was approved by the Institutional Review Board of the China Medical University Hospital. Informed consent was signed by relatives. Tissue sampling was based on individual infarct topography, which in each case was determined on the basis of cerebrovascular anatomy and the most recent magnetic resonance image (MRI) scan. On autopsy, brain areas with variable degrees of infarction were identified macroscopically and approximately 1 cm^3^ cortical samples, including subcortical white matter, were dissected and fixed with formalin prior to embedding in paraffin or frozen at −70°C until analysis, as described previously [1]. Immunostaining of brain samples was performed using specific antibody of PACAP38 (1:200, Santa Cruz Biotechnology, Santa Cruz, CA, http://www.scbt.com), CD34 (1:100, BD Pharmingen, San Diego, CA, http://www.bdbiosciences.com/index[lowen]us.shtml), PAC1 (1: 300, Santa Cruz Biotechnology), βIII-tubulin (Tuj-1) (1:200; Chemicon, Temecula, CA, http://www.chemicon.com), microtubule-associated protein 2 (MAP-2, 1:200; BM). Samples from corresponding areas of the contralateral or noninfarcted hemispheres and from the control brains were processed in a similar way. The extent of PACAP38^+^ cell immunoreactivity was measured as the number of cells per square millimeter (cells per mm^2^).

### In Vivo Ischemia/Reperfusion Brain Model

Adult male Sprague-Dawley rats (weight 250–300 g) were used in this study. All surgical procedures were performed using sterile/aseptic techniques in accordance with Institutional guidelines. Rats were anesthetized with chloral hydrate (0.4 g/kg i.p.) and subjected to cerebral ischemia. Ligation of the right middle cerebral artery (MCA) and bilateral common carotids arteries (CCAs) was performed by methods described previously [1]. The CCAs were clamped with nontraumatic arterial clips. The right MCA was ligated with a 10-0 nylone suture. After 90 minutes of ischemia, the sutures on the MCA and the arterial clips on the CCAs were removed to allow reperfusion. Core body temperature was monitored with a thermistor probe (Hewlett-Packard Model 21090A probe, Hewlett-Packard Company, Andover, MA) and maintained at 37°C with a heating pad during anesthesia. After recovery from anesthesia, rat body temperature was maintained at 37^o^C with a heat lamp.

In addition, conditional *HIF-1α* knockout mice (HIF-1α KO mice carrying a loxP-flanked allele of HIF-1α, a kind gift from Dr. Johnson [21]). HIF-1α disruption in the HIF-1α KO mice was induced by feeding doxycycline at a dose of 2 mg/mL in 5% (w/v) sucrose solution from embryonic day 15 to postnatal day 1. They were also anesthetized with chloral hydrate (0.3 g/kg, i.p.) and subjected to right MCA ligation and bilateral CCAs clamping for 120 minutes, as described above with modification.

### 2-Methoxyestradiol Treatment In Vivo and In Vitro

2-Methoxyestradiol (2-ME2, Sigma) was dissolved in dimethyl sulfoxide (DMSO) to obtain a 10 mmol/L stock solution. For in vivo experiments, the whole procedure was as previously described [22]. Experimental rats were treated with an intraperitoneal injection of a liposomal preparation (di-oleoyl-phosphotidylcholine; Avanti Polar Lipids) of 2-ME2 (20 mg/mL) in a different concentration (50, 100, or 150 mg/kg) for 3 consecutive days before and after the onset of cerebral ischemia. For in vitro experiments with 2-ME2 treatment, primary cortical cultures (PCCs) were pretreated with different concentrations of 2-ME2 (0.1 μM, 1 μM, and 10 μM) for 16 hours as previously described [23].

### Total Protein Extraction for Western Blotting and ELISA

Experimental animals were decapitated at 4 hours, 12 hours, 3 days, and 7 days after reperfusion with 90 minutes MCA ligation. Three rats without MCA ligation were used as normal controls. Samples of ischemic cerebral cortex were taken from the peripheral region of infarcted brains (penumbral area). Western blot analysis was performed on these samples. Briefly, ischemic brain tissue was homogenized and lysed in the buffer containing 320 mM sucrose, 5 mM HEPES, 1 μg/mL leupeptin, and 1 μg/mL aprotinin. Lysates were centrifuged at 13,000*g* for 15 minutes. The resulting pellet was resuspended in sample buffer [62.5 mM Tris-HCl, 10% glycerol, 2% SDS, 0.1% bromophenol blue, and 50 mM dithiothreitol (DTT)] and subjected to SDS-polyacrylamide gel (4%–12%) electrophoresis. Then, the gel was transferred to a Hybond-P nylon membrane. This was followed by incubation with appropriately diluted antibodies of PACAP38 (1:200; Invitrogen, Carlsbad, CA, http://www.invitrogen.com), HIF-1α (1:200; Novus Biologicals), HIF-2α (1:200; Novus Biologicals), PAC1 (1: 300, Santa Cruz Biotechnology), PrP^C^ (1:300, Santa Cruz Biotechnology), α6-integrin (1:200, Chemicon), platelet/endothelial cell adhesion molecule 1 (PECAM-1) (1:200; Santa Cruz Biotechnology), selectin (1:200; Santa Cruz Biotechnology), CXCR4 (1:200; R&D Systems, Minneapolis, MN, http://www.rndsystems.com), CC chemokine receptor 3 (CCR3) (1:200; R&D Systems), CCR4 (1:200; R&D Systems), β1-integrin (1:200; Chemicon), β2-integrin (1:200; Chemicon), junctional adhesion molecular A (JAM-A) (1:200; Millipore, Billerica, MA, http://www.millipore.com), junctional adhesion molecular C (JAM-C) (1:200; Millipore), lymphocyte function-associated antigen 1 (LFA-1) (1:200; Millipore), intercellular adhesion molecule (ICAM) (1:200; Millipore), vascular cell adhesion molecule (VCAM-1) (1:200; Millipore), vascular endothelial (VE)-cadherin (1:200; Millipore), CD99 (1:200; Millipore), focal adhesion kinase (FAK) (1:200; Millipore), stress-induced-phosphoprotein 1 (STI-1) (1:200; Santa Cruz Biotechnology), and β-Actin (dilution 1:2,000; Santa Cruz Biotechnology). Expression of apoptosis-related proteins (Bcl-2, Bcl-xL, Bax, and Bad) in the right cortex and striatum region was also examined [24]. Membrane blocking, primary and secondary antibody incubations, and chemiluminescence reactions were conducted for each antibody individually according to the manufacturer's protocol. The intensity of each band was measured using a Kodak Digital Science 1D Image Analysis System (Eastman Kodak). The ratio of band intensity of each protein in Western blots in comparison with the internal control was calculated. In addition, PACAP38 levels were measured by direct ELISA using goat-polyclonal PACAP antibody (1:1,000; Santa Cruz Biotechnology) and peroxidase-labeled donkey anti-goat IgG (1:2,000, Santa Cruz Biotechnology). Optical density was measured using a spectrophotometer (Molecular Devices, Union City, CA, http://www.moleculardevices.com), and standard curves were generated with the program SOFTmax (Molecular Devices).

### Measurement of HIF-1α Activity by ELISA

To measure the active HIF-1α, 50 μg nuclear extracts were incubated with biotinylated double stranded oligonucleotide containing a consensus HIF-1α binding site from Duo-set ELISA mouse active HIF-1α kit (R&D Systems) according to the manufacturer's instructions. The activity of HIF-1α was expressed by OD (450–540 nm) as previously described [23]. The experiments were carried out in triplicate and each experiment was repeated three times unless otherwise mentioned.

### Immunohistochemical Analysis of Rat Brain

Experimental rats were re-anesthetized with chloral hydrate (0.4 g/kg i.p.) and were decapitated at 4 hours, 12 hours, 3 days, and 7 days after cerebral ischemia. Three rats without MCA ligation were used as normal controls. Rat brains were fixed by transcardial perfusion with saline, followed by perfusion and immersion in 4% paraformaldehyde as previously described [7]. A series of adjacent 6-μm-thick sections were cut by cryostat from each tissue block in the coronal plane, stained with H&E, and analyzed by light microscopy (Nikon, E600).

To identify the expression of cell type-specific markers in PACAP38^+^ cells, double immunofluorescence was performed as previously described [1]. Each coronal section was first stained with primary PACAP38 antibody (1:100, Santa Cruz Biotechnology), followed by treatment with specific antibodies: glial fibrillary acidic protein (GFAP) (1:400, Sigma), Von Willebrand factor (vWF) (1:400; Sigma), Tuj-1 (1:200; Chemicon), MAP-2 (1:200; BM), CD34 (1:100, BD Pharmingen), PAC1 (1:300, Santa Cruz Biotechnology), PrP^C^ (1:300, Santa Cruz Biotechnology), α6-integrin (1:200, Chemicon), Laminin (1:500, Sigma), and HIF-1α (1:200; R&D Systems). The tissue sections were analyzed with a Carl Zeiss LSM510 laser-scanning confocal microscope. Fluorescein isothiocyanate (FITC) (green, 1:500; Jackson Immunoresearch Laboratories, West Grove, PA, http://www.jacksonimmuno.com), Cy3 (red, 1:500; Jackson Immunoresearch), and Alexa Fluor 408 (blue, 1:1,000; Invitrogen) fluorochromes on the immunofluorescence-labeled slides were excited by laser beam at 488 nm, 543 nm, and 680 nm, respectively. PACAP38, labeled with Cy3 (red) or FITC (green) or Alexa Fluor 680 (blue, 1:1,000; Invitrogen) fluorochromes, and cell-type-specific markers of neuronal nuclear (Neu-N), MAP-2, vWF, GFAP, and HIF-1α, labeled with Cy3 (red) or FITC (green) fluorochromes were double immunostained in order to demonstrate their colocalization in one cell under laser-scanning confocal microscopy. For quantification, distance of cell nuclei (GFP^+^ cells) from blood vessels (laminin^+^) was quantified with an automated system, described previously [25].

### Cerebral Ischemic Animal Model Treated with PACAP38

The cerebral ischemic animal model was established as described above. Experimental rats were injected intraperitoneally with different dosage of PACAP38 (0.1, 1, and 10 μg/kg, Sigma Aldrich) at 4 hours after MCA ligation for five consecutive days. In addition, two therapeutic group of PACAP38 (10 μg/kg) and vehicle control were progressed for further investigation. Core body temperature was monitored with a thermistor probe and maintained at 37°C with a heating pad during anesthesia. For the blocking experiment, 100 mg/kg of a broad, class-specific metalloproteinase inhibitor (GM6001; Chemicon) was injected intraperitoneally for 4 consecutive days as previously described [26]. In addition, specific PAC1 antagonist PACAP (6–38) (Bachem, CA) 10 μg/kg was administered intraperitoneally for 4 consecutive days as previously described with modification [27].

### Triphenyltetrazolium Chloride Staining

Three days after cerebral ischemia, animals were intracardially perfused with saline. The brain tissue was removed, immersed in cold saline for 5 minutes, and sliced into 2.0-mm-thick sections (seven slices per rat). The brain slices were incubated in 20 g/L triphenyltetrazolium chloride (TTC; Research Organics), dissolved in saline for 30 minutes at 37^o^C, and then transferred into a 5% formaldehyde solution for fixation. The area of infarction in each slice was measured with a digital scanner, as described previously [1].

### Neurological Behavioral Measurements

Behavioral assessments were performed 3 days before cerebral ischemia and 72 hours after cerebral ischemia. The tests measured (a) body asymmetry and (b) locomotor activity [7]. Furthermore, grip strength was analyzed using Grip Strength Meter (TSE-Systems) as previously described with modification [1]. The baseline-test scores were recorded in order to normalize those taken after cerebral ischemia. The elevated body swing test was used to assess body asymmetry after MCA ligation and evaluated quantitatively as previously described [7]. Initially, animals were examined for lateral movement by suspending their bodies by their tails. The frequency of initial head swing contralateral to the ischemic side was counted in 20 continuous tests and was normalized, as follows: % recovery = [1 – (lateral swings in twenty tests – 10)/10 × 100%. Locomotor activity: Rats were subjected to VersaMax Animal Activity monitoring (Accuscan Instruments) for about 2 hours for behavioral recording. The VersaMax Animal Activity monitoring contained 16 horizontal and 8 vertical infrared sensors spaced 87 cm apart. The vertical sensors were situated 10 cm from the floor of the chamber. Motor activity was counted as the number of beams broken by a rat movement in the chamber. Three vertical parameters defined in the manufacturer's menu option were calculated over 2 hours at night: (a) vertical activity, (b) vertical movement time, and (c) number of vertical movements. In grip strength analysis, ratio of improvement in grip strength was measured on each forelimb separately and was calculated as the ratio between the mean strength out of 20 pulls of the side contralateral to the ischemia and the ipsilateral side [1]. In addition, the ratio of grip strength post-treatment and baseline were also calculated, and changes were presented as a percentage of baseline value.

### Measurement of Infarct Size Using MRI

MRI was performed on rats under anesthesia in an imaging system (R4, General Electronics) at 3.0 T. Brains were scanned in six to eight coronal image slices, each 2 mm thick without any gaps. *T*_2_-weighted imaging (T2WI) pulse sequences were obtained with the use of a spin-echo technique (repetition time, 4,000 milliseconds; echo time, 105 millisecond) and were captured sequentially for each animal at 1, 7, and 28 days after cerebral ischemia. To measure the infarction area in the right cortex, we subtracted the noninfarcted area in the right cortex from the total cortical area of the left hemisphere [1]. The area of infarct was drawn manually from slice to slice, and the volume was then calculated by internal volume analysis software (Voxtool, General Electric).

### [^18^F]fluoro-2-deoxyglucose Positron Emission Tomography Examination

To assess the metabolic activity and synaptic density of brain tissue, experimental rats were examined using microPET (Positron emission tomography) scanning of [^18^F]fluoro-2-deoxyglucose (FDG) to measure relative metabolic activity under the protocol previously described [28]. In brief, ^18^F was produced by the ^18^O(p, n)^18^F nuclear reaction in a cyclotron at China Medical University and Hospital, Taiwan, and ^18^F-FDG was synthesized as previously described [29] with an automated ^18^F-FDG synthesis system (Nihon Kokan). Data were collected with a high-resolution small-animal PET (microPET, Rodent R4, Concorde Microsystems) scanner. The system parameters have been described previously by Carmichael et al. [30]. After 1 week of each treatment, animals were anesthetized with chloral hydrate (0.4 g/kg, i.p.), fixed in a customized stereotactic head holder, and positioned in the microPET scanner. Then the animals were given an intravenous bolus injection of ^18^F-FDG (200–250 μCi per rat) dissolved in 0.5 mL of saline. Data acquisition began at the same time and continued for 60 minutes using a 3D acquisition protocol. The image data acquired from microPET were displayed and analyzed by Interactive Data Language ver. 5.5 (Research Systems) and ASIPro ver. 3.2 (Concorde Microsystems) software. FDG-PET images were reconstructed using a posterior-based three-dimensional iterative algorithm [31] and overlaid on MR templates to confirm anatomical location [32]. Coronal sections for striatal and cortical measurements represented brain areas between 0 and +1 mm from bregma, and thalamic measurements represented brain areas between −2 and −3 mm from bregma, as estimated by visual inspection of the unlesioned side. The relative metabolic activity in regions of interest of the striatum was expressed as a percentage deficit as previously described with modification [30].

### Preparation of Stem Cells Culture

BMDCs were collected from rat femoral veins (5 mL) mobilized with recombinant human granulocyte colony-stimulating factor (CSF, Kirin, Tokyo, Japan) at 50 μg/kg per day subcutaneously for 5 consecutive days. The mononuclear cells (MCs) were separated by Ficoll-Paque (1:3 dilution, StemCell Technologies, Vancouver, BC, Canada, http://www.stemcell.com). CD34^+^ BMDCs were separated from 2 × 10^6^ MCs by a magnetic bead separation method (MACS; Miltenyi Biotec, Gladbach, Germany, http://www.miltenyibiotec.com) according the manufacturer's instructions. Subsequently, CD34^+^ BMDCs (purity > 95%, 10^6^ cells per mL) were cultured for 72 hours in medium (StemSpan H3000 and Cytokine Cocktail, StemCell Technologies) at 37°C in a humidified atmosphere of 5% CO_2_ /95% air and antibiotics and prepared for experiment. In bromodeoxyuridine (BrdU) labeling and immunocytochemistry, the cells were pulsed with 10 μM BrdU for 4 hours and fixed with 4% paraformaldehyde for 20 minutes as previously described [33]. In brief, DNA was denatured by treatment with 2.5 N HCl for 20 minutes at room temperature followed by 0.1 M boric acid treatment to neutralize the cells. Incorporated BrdU was detected with a mouse monoclonal anti-BrdU antibody (1:50, BD Biosciences) that was incubated with the cells overnight. The percentage of BrdU-positive cells was determined by counting under a phase contrast microscope and at least 500 cells per sample were scored.

### Transwell Migration Assays

Enhanced migration of BMDCs by PACAP38 treatment was assessed as described previously with modifications [34]. In brief, BMDCs treated with PACAP38 (1, 10, and 100 nM) [35] were placed in 100 μL in the upper chamber (transwell: 6.5-mm diameter, 5.0-mm pore size) according to manufacturer's instructions (Costar, #3421). We used SDF-1α (100 ng/mL, R&D System, positive control) in the lower chambers. For the PAC1 receptor blocking study, PACAP (6–38) (10 μM, Bachem, Switzerland) was also added to the lower wells [36]. The assays were conducted over a 4-hour incubation period at 37°C in a 5% CO_2_ incubator. Because almost all cells stay at the lower side of the membrane after migration, quantification can be performed by simply counting these cells. Adhered cells at the lower side of the membranes were counted under the microscopy as previously described [34]. For assessing migrated CD34^+^ cells, cells were collected from the bottom of a transwell and assessed by flow cytometry as described previously [37].

### Gene Silencing with RNA Interference

Specific knockdown was achieved by lentiviral delivery of short hairpin RNA (shRNA) for PACAP38 (LV-PACAP-sh; sc-39531-V, Santa Cruz Biotechnology), shRNA for HIF-1α (LV-HIF-1α-sh; sc-35562-V, Santa Cruz Biotechnology), shRNA for HIF-2α (LV-HIF-2α-sh; sc-35316-V, Santa Cruz Biotechnology), Lenti-PrP^C^ shRNA (LV-PrP^C^-sh, sc-36318-V, Santa Cruz Biotechnology), Lenti-α6-integrin shRNA (LV-α6-integrin-sh, sc-43129-V, Santa Cruz Biotechnology), and the control scramble shRNA (LV-control-sh; sc-108080, Santa Cruz Biotechnology) under manufacture's instruction.

### Lentiviral Constructs of PACAP38, PACAP38-Flag, HIF-1α, and HIF-2α

The lentiviral constructs were generated by cotransfection of human kidney derived 293T cells with three plasmids using the calcium phosphate method as previously described with modification [38]. In the transducing vector, an expression cassette with the Rev responsive element and the elongation factor-1 (EF-1α) promoter are used to direct the expression of PACAP38 (PACAP38 cDNA, SC110820, OriGene), mouse HIF-1α and HIF-2α (clone ID 4019056 and 5032291, Thermo) [39] and green fluorescent protein (GFP cDNA; Clontech, Palo Alto, CA, http://www.clontech.com). Lentiviral vector particles were generated by transient cotransfection of 293T cells with the lentiviral shuttle plasmid from TRIP GFP plasmid vector [40], an HIV-1-derived packaging plasmid, and a VSV-G envelope expressing plasmid. Two days after transfection, lentiviral constructs (LV-PACAP38/-Flag, LV-HIF-1α, LV-HIF-2α, or LV-GFP) were harvested in the culture medium and concentrated by ultracentrifugation. Viral titers were quantified by using HIV-1 p24 antigen assay (Beckman Coulter) according to the manufacturer's instructions. The p24 concentration was used to determine the vector dose (expressed in nanograms) administered in the various in vitro and in vivo experiments. The lentiviral titers were determined by infection of 293T cells seeded in six-well plates at 1 × 10^5^ cells per well the day before infection with serial dilution of the concentrated viral stock. After overnight incubation, the culture medium was changed and the cells incubated for 2 more days. GFP fluorescent cells were identified by fluorescent microscopy or by a fluorescent activated cell sorter. Titers ranged from 10^8^ to10^9^ infectious units per mL.

### Lentiviral Vector Administration In Vivo and In Vitro

Intracerebral administration was performed in animals under chloral hydrate anesthesia to inject with 1 × 10^9^ viral units of LV-PACAP38-shRNA, LV-PACAP38/-Flag, LV-GFP, or control shRNA (5 μL) through a 26-gauge Hamilton syringe (Hamilton Company) into three cortical areas, 3.0–5.0 mm below the dura. The approximate coordinates for these sites were 1.0–2.0 mm anterior to the bregma and 2.5–3.0 mm lateral to the midline, 0.5–1.5 mm posterior to the bregma and 3.5–4.0 mm lateral to the midline, and 3.0–4.0 mm posterior to the bregma and 4.5–5.0 mm lateral to the midline. The needle was retained in place for 5 minutes after each injection and a piece of bone wax was applied to the skull defects to prevent leakage of the injected solution. To assess for transgene expression after intracerebral injection of lentiviral vector, animals received intracerebral injection of lentiviral particles were then killed for histological purposes and western blot quantification of PACAP38 production in vivo. In in vitro lentiviral vectors transduction, cell culture was plated in 10-cm dishes at a density of 1 × 10^5^ cells in 5 mL medium per dish. Transductions were carried out in the presence of 8 μg/mL polybrene at m.o.i. of 5 or 25 for each vector. After incubation for 24 hours, the transduction medium was replaced with fresh original medium for each cell.

#### Gel Zymography

The culture supernatant containing equal amounts of protein was loaded onto a 10% SDS-polyacrylamide gel containing gelatin (Bio-Rad, Hercules, CA, http://www.bio-rad.com). After electrophoresis, gels were washed in 5% Triton X-100 and then incubated in matrix metalloprotease (MMP) assay buffer (Bio-Rad). Bands were visualized with Coomassie Brilliant Blue and destained in 30% methanol with 10% acetic acid.

### Preparation of Transgenic GFP-Chimeric Mice

In order to verify the enhancement of bone marrow stem cell mobilization and homing into brain, a bone marrow niche sample was removed from the long bones of adult male donor GFP mice as previously reported [41]. Both ends of the femur and tibia were penetrated using a syringe with a 25-gauge needle, and the marrow was flushed out with sterile saline. Total marrow from one femur was diluted to 1 mL then strained through a 30-µm Spectramesh (Fisher Scientific). Before bone marrow transplantation, recipient wild type (C57BL/6 mice-*PAC1^+/^^+^* mice) and *PAC1^−^^/^^−^* mice [a generous gift provided by DKFZ Dr. Schutz [42]] underwent whole body gamma irradiation with ^137^Cs using a Gammacell 40 irradiator (MDS Nordion). A total dose of 9 Gy (900 rads) was administered to ablate the whole bone marrow. The mice received rescuing bone marrow transplantations within 24 hours of irradiation. Donor bone marrow was injected into the recipient animal's tail vein as an 80 µL cell suspension containing 3 × 10^6^ cells. At 3 weeks after transplantation, mice were anesthetized with chloral hydrate (0.3 g/kg, i.p.) and subjected to right MCA ligation and right CCAs clamping for 120 minutes, as previously described with modification [24]. Then, experimental mice were injected intraperitoneally with PACAP38 (50 μg/kg) and vehicle control.

### Angiogenic Evaluation by FITC-Dextran Perfusion and CD31 Immunohistochemistry

In order to examine the blood vessels, cerebral microcirculation was analyzed by administering the fluorescent plasma marker (FITC-dextran, Sigma) intravenously to rats and observing them under fluorescent microscopy (Carl Zeiss, Axiovert 200M), as previously described [43]. In addition, to quantify the cerebral blood vessel density and examine the vascular remodeling by macrophage, experimental rats were anesthetized with chloral hydrate and perfused with saline. Histological sections (6 μm) were stained with specific antibody to CD-31 (1:100, BD Pharmingen) conjugated with Cy-3 or FITC (1:500, Jackson Immunoresearch). The number of blood vessels was determined as previously described [44].

### Measurement of Cerebral Blood Flow

Experimental rats were positioned in a stereotaxic frame and baseline local cortical blood flow (bCBF) was monitored after cerebral ischemia with a laser doppler flowmeter (LDF monitor, Moor Instrutments, Axminster, U.K.) in anesthetized state (chloral hydrate) as previously described [45]. In brief, cerebral blood flow (CBF) values were calculated as percentage increase compared to the bCBF.

### In Vitro PCCs Preparation

PCCs were prepared from the cerebral cortex of gestation day 17 embryos from wild type (C57BL/6 mice-*PAC1^+/^^+^* mice) and *PAC1*^−/−^ mice as described previously [46]. PCCs were maintained under serum-free conditions in neurobasal medium (Invitrogen), supplemented with B-27 supplement (2%; Invitrogen), glutamine (0.5 mM; Sigma), glutamate (25 mM; Sigma), penicillin (100 U/mL), and streptomycin (100 mg/mL; Invitrogen Corp.). At 4 days in vitro, half of the medium was removed and replaced with fresh medium without glutamate, as indicated by the manufacturer. The cultures were maintained in a humidified incubator at 37°C with 5% CO_2_. At 7 days in vitro, PCCs were used for experimentation.

### Hypoxia Procedure

PCCs (1 × 10^5^ per mL) cultured at 37°C in 5% CO_2_-humidified incubators were treated in normoxic (21% O_2_) or hypoxic conditions (1% O_2_) for different time points as previously described [47]. Hypoxic cultures were cultivated in a two-gas incubator (Jouan, Winchester, VA) equipped with an O_2_ probe to regulate N_2_ levels. Cell number and viability were evaluated using trypan blue exclusion assay.

### Immunocytochemical and Western Blot Analysis of PCCs

Following hypoxia (1% O_2_ for 12 hours), PCCs were collected for PACAP38 immunostaining at each time point, cell cultures were washed with phosphate buffered saline (PBS) and fixed for 30 minutes at room temperature in 4% paraformaldehyde. After being washed with PBS, the fixed cultured cells were treated for 30 minutes with blocking solution (10 g/L bovine serum albumin (BSA), 0.03% Triton X-100, and 4% serum in PBS). PCCs were incubated overnight at 4°C with an antibody against PACAP38 (1:100, Santa Cruz Biotechnology) and then rinsed three times in PBS. The extent of PACAP38^+^ cell immunoreactivity was measured as the number of cells per square millimeter (cells per mm^2^). PCCs expression of PACAP38 were measured by Western blot analyses using appropriately diluted antibodies to PACAP38 (1:100, Santa Cruz Biotechnology) as mentioned above.

### Terminal Deoxynucleotidyl Transferase-Mediated Digoxigenin-dUTP Nick-End Labeling Histochemistry

To detect cellular apoptosis, a terminal deoxynucleotidyl transferase-mediated digoxigenin-dUTP nick-end labeling (TUNEL) staining Kit (DeadEnd Fluorimetric TUNEL system, Promega, Madison, WI, http://www.promega.com) was used for the TUNEL assay. Twenty-four hours after oxygen glucose deprivation (OGD), the cells were fixed with 4% paraformaldehyde in PBS for 20 minutes at 4°C and subjected to permeabilization for 20 minutes at room temperature with 0.1% sodium citrate containing 0.1% Triton X-100. The fixed and permeabilized PCCs were labeled with the TUNEL reaction mixture for 60 minutes at 37°C. The nuclei of these PCCs were counterstained with DAPI. The percentage of TUNEL labeling was expressed as the number of TUNEL-positive nuclei divided by the total number of nuclei stained with DAPI [48].

### Chromatin Immunoprecipitation Assay

To demonstrate the binding of HIF-1α protein to the promoter of PACAP, the chromatin immunoprecipitation (ChIP) assay was performed with a commercial kit (Upstate Biotechnology) using the manufacturer's protocol with minor adjustments. The PCCs were grown and incubated in air or 1% O_2_ for 4 hours, and formaldehyde was added directly to culture medium to a final concentration of 1% followed by incubation for 20 minutes at 37°C as previously described [49]. DNA-protein complexes were isolated on salmon sperm DNA linked to protein A agarose beads and eluted with 1% SDS, and 0.1 M NaHCO_3_. Crosslinking was reversed by incubation at 65°C for 5 hours. Proteins were removed with proteinase K, and DNA was extracted with phenol/chloroform, redissolved, and polymerase chain reaction (PCR)-amplified with PACAP promoter primers, sense: 5′-GAGGGACTAGGATGCTGACG-3′; and antisense: 5′-TGTTGCGCTCCG ATTTTTAT-3′.

### Generation of Promoter Constructs, Transient Transfection, and Reporter Gene Assays

A luciferase construct containing the 5′-flanking region of the PACAP gene promoter was a kind gift from Miyata et al. [50]. This luciferase reporter product PPR1 was further subcloned into the BamHI and SphI sites of the pGL3-basic vector (Promega) which contained one real HRE, and the generated plasmid was designated pPACAP-luc1. One additional PACAP promoter constructs (pPACAP-luc2) using the same downstream primer as for pPACAP-luc1 did not contain the HRE. In the pPACAP-mutHRE construct, the putative HRE of pPACAP-luc1 was replaced from 5′-ACGTG-3′ to 5′-AAAAG-3′ using the QuikChange Site-Directed Mutagenesis Kit (Stratagene, La Jolla, CA, http://www.stratagene.com). All constructs were verified by DNA sequencing. 3T3 NIH cells at about 90% confluence in 24-well plates were transiently transfected with reporter plasmid (0.5 μg) using Lipofectamine 2000 (Invitrogen) according to the manufacturer's directions. To correct for variable transfection efficiency, cells were cotransfected with the pRL-SV40 vector (0.05 μg) encoding the *Renilla* luciferase gene. Transfected cells were allowed to recover for 24 hours in fresh medium, and then subjected to 1% O_2_ for 8 hours. Cells were lysed and luciferase activity was determined with a multiwell luminescence reader (Molecular Devices), by using the Dual-Luciferase Reporter Assay System (Promega).

### Statistical Analysis

Observers were blind to the experimental conditions of each measurement. Results are expressed as mean ± SEM. The behavioral scores were evaluated for normality. We used one-way or two-way ANOVA with appropriate post hoc Newman-Keuls testing to evaluate mean differences between different groups with different treatments. A value of *p* < .05 was considered as significant.

## Results

### PACAP38 is Upregulated in the Ischemic Brains from Human Patients and Animals

**Figure 1:**
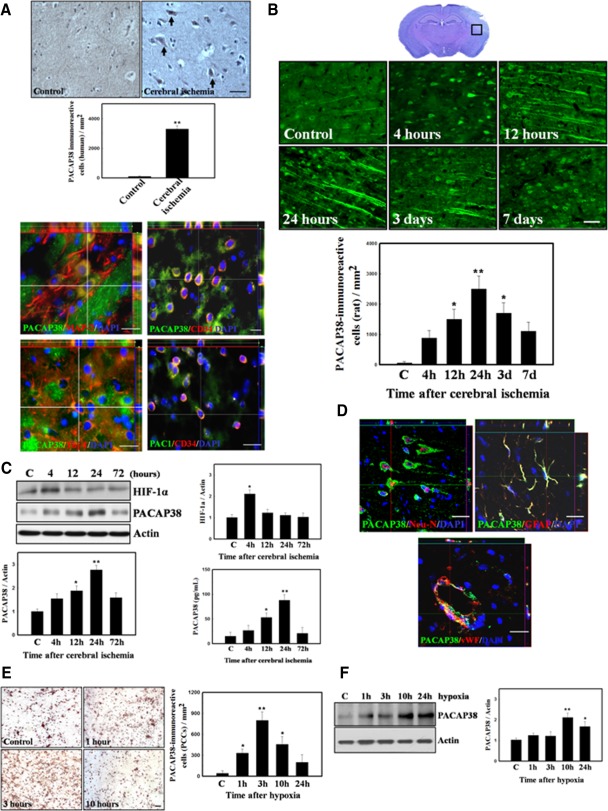
Hypoxia/ischemia-induced upregulation of PACAP38 expression in human and rat brain and PCCs. (A): Representative figures from patients 1 and 3 days after a stroke onset (upper panel). In immunohistochemical analysis of the penumbral area for PACAP38 (black arrows), quantitative measurement showed significantly increased numbers of PACAP38^+^ cells in the brains of patients after cerebral infarction as compared with controls. By 3D image, MAP-2^+^ and Tuj-1^+^ neuronal cells were costained with PACAP38 (left lower panel). In addition, some PACAP38^+^CD34^+^ and PAC1^+^CD34^+^ cells were dispersed over the penumbric region (right lower panel). (B): The square (□) indicates the representative area of cerebral ischemia for which immunoreactivity for PACAP38 is shown. These areas include the penumbral and perivascular regions, which were examined at different time points (4, 12, and 24 hours and 3 and 7 days) after cerebral ischemia. Quantitative analysis of PACAP38 immunoreactivity over time is shown (C, control). (C): Western blot analysis of HIF-1α and PACAP38 expression for ischemic brain, data from Western blots was quantified and is shown with respect to control expression. Quantitative data from ELISA of PACAP38 are shown. (D): Double immunofluorescence (3D images) showed that cells expressing PACAP38 in the penumbra area colocalized with the specific markers Neu-N, GFAP, and vWF. (E): Immunohistochemistry of PCCs for PACAP38 under hypoxic conditions, quantitative data from PACAP38^+^ cells are shown. (F): Western blotting for PACAP38 expression in PCCs after hypoxia treatment, quantitative data from western blots are shown. The mean ± SEM is shown. *, *p* < .05 and **, *p* < .01 vs. control. Scale bar = 50 μm. Abbreviations: DAPI, 4′,6-diamidino-2-phenylindole dihydrochloride; GFAP, glial fibrillary acidic protein; HIF-1α, hypoxia-inducible factor-1α; MAP-2, microtubular associated protein-2; Neu-N, neuronal nuclear; PACAP38, pituitary adenylate cyclase-activating peptide 38; PAC1, PACAP type 1 receptor; PCC, primary cortical culture; Tuj-1, βIII-tubulin; vWF, Von Willebrand factor.

To determine whether cerebral ischemia increases PACAP38 expression, we performed PACAP38 immunostaining in human and rat brain after stroke. Human brain samples (*n* = 4) from patients who died at various times after stroke and rat brains at different times (4 hours to 7 days) after focal cerebral ischemia were processed for PACAP38 immunoreactivity. Increased number of PACAP38^+^ cells was noted in the penumbral region in the brain samples from patients who died 1–3 days after cerebral infarction as compared with the control (Fig. 1A). PACAP38^+^ cells coexpressed the neuronal marker proteins MAP-2 and Tuj-1 (Fig. 1A). Interestingly, some PACAP38^+^ and PAC1^+^ cells costained with CD34^+^-expressing cells, suggesting that circulating CD34^+^-expressing cells may acquire PACAP38 and PAC1 expression upon translocation to ischemic brain regions (Fig. 1A). Similar to the findings in stroke patients, increase in number of PACAP38^+^ cells were also detected in the ipsilateral cortex near the infarct boundary in rat brains with a time-dependent manner, reaching the peak at 24 hours after cerebral ischemia (Fig. 1B).

### HIF-1α Induced PACAP38 Expression by Binding to the PACAP38 Promoter

To determine whether HIF-1α and PACAP38 expression was increased in stroke rats, PACAP38 levels in brains (cortical region and striatum) from rats after cerebral ischemia were measured using Western blotting and ELISA and compared with nonischemic rats. Cerebral ischemia in rat brains caused a time-dependent increase in HIF-1α and PACAP38 expression (Fig. 1C). ELISA also revealed significantly increased PACAP38 protein level in ischemic rats as compared with nonischemic controls (Fig. 1C). To identify cell types expressing PACAP38 after cerebral ischemia, double immunofluorescence staining under laser scanning confocal microscopy was used to observe the expression patterns of PACAP38 in colocalization with neuronal marker of Neu-N, glial marker of GFAP, and endothelial marker of vWF in the ischemic brains. The PACAP38 was strongly coexpressed with Neu-N, GFAP, and vWF in ischemic areas (Fig. 1D), suggesting brain ischemia upregulated PACAP38 in neurons, glia, and endothelial cells. To examine the effect of hypoxia on PACAP38 expression in PCCs, cells were placed under in vitro hypoxic conditions for 1, 3, 10, or 24 hours. PACAP38 protein expression was also increased after hypoxic stress (Fig. 1E, 1F).

**Figure 2:**
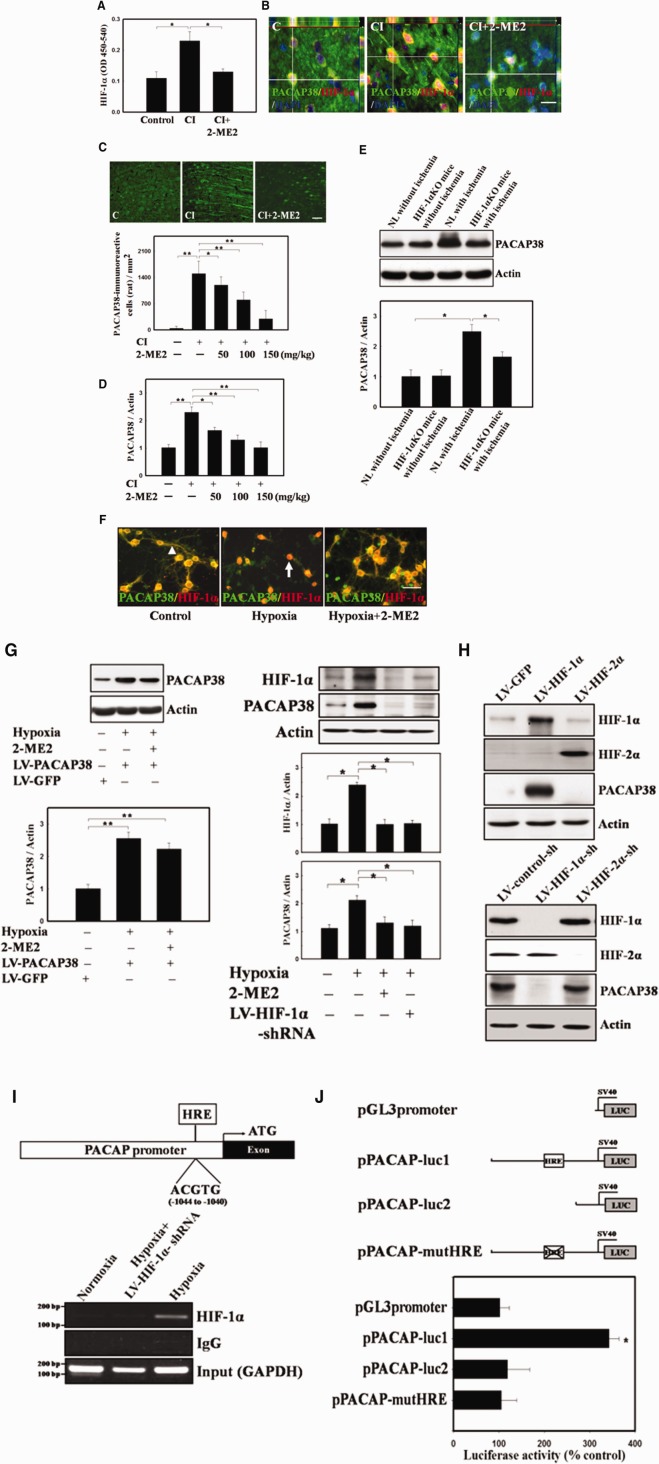
Upregulation of PACAP38 after CI and hypoxia via induction of HIF-1α in vivo and in vitro. (A): Nuclear HIF-1α activity increased in the brains of CI rats and was blocked by intraperitoneal administration of 2-ME2 (100 mg/kg). (B): Increasing numbers of PACAP38^+^ cells colocalizing with HIF-1α^+^ cells in the ischemic penumbral area were abolished by injection of 2-ME2 (C, nonischemic control). 2-ME2 injection inhibited the HIF-1α translocation into nucleus in the ischemic brain. (C): PACAP38 immunoreactivity decreased in a dose-dependent manner in the presence of 2-ME2 after CI (C, nonischemic control). (D): Western blot analysis of PACAP38 in the ischemic rat brain, quantitative data from Western blots are shown. (E): In HIF-1α KO mice, CI did not significantly induce PACAP38 upregulation as compared with NL. Quantitative data from Western blots are shown. (F): Double immunofluorescence for HIF-1α and PACAP38 in primary cortical cultures (PCCs) in the absence and presence of a 16-hour pretreatment with 2-ME2 (10 µM) before hypoxia (nuclei or perinuclear area, arrow; cytosol, neurites arrowhead). (G): 2-ME2 specifically inhibited HIF-1α-induced PACAP38 expression but not lentiviral (LV-PACAP38) infection-induced PACAP38 expression (left panel). In addition, PACAP38 expression was downregulated by LV-HIF-1α-sh. (LV-GFP and LV-control-sh) were used as a nonspecific control under hypoxic conditions, right panel). (H): Modulation of PACAP38 expression in PCCs was found in genetic targeting of HIF-1α via lentiviral transduction (LV-HIF-1α or LV-HIF-1α-sh) at 1 hour after hypoxia compared to that of HIF-2α by western blot. (I): Schematic representation of the 5′-flanking PACAP genomic region with the promoter and first exon. The hypoxic-responsive element (HRE) binding site corresponds to nucleotides −1,044 to −1,040 (relative to putative transcription start site +1). Binding of HIF-1α to the PACAP promoter was detected with a chromatin immnuoprecipitation assay in PCCs subjected to hypoxia (LV-HIF-1α-shRNA was used as a negative control). (J): A luciferase reporter assay in hypoxia-treated cells transfected with pPACAP-luc1 as compared with a control construct (pPACAP-luc2) and an HRE-mutant construct (pPACAP-mutHRE). *n* = 8 per group. The mean ± SEM is shown. *, *p* < .05 and **, *p* < .01 vs. control. Scale bar = 50 μm. Abbreviations: CI, cerebral ischemia; DAPI, 4′,6-diamidino-2-phenylindole dihydrochloride; GFP, green fluorescent protein; HRE, hypoxic responsive element; HIF-1α, hypoxia inducible factor 1α; KO, knockout; 2-ME2, 2-Methoxyestradiol; NL, normal littermate; PACAP38, pituitary adenylate cyclase-activating peptide 38.

We next examined whether PACAP38 upregulation after ischemia or hypoxia is mediated through HIF-1α activation. Cerebral ischemia increased the expression of activated nuclear HIF-1α, which was inhibited by 2-ME2 (100 mg/kg) injection (Fig. 2A). Double immunofluorescence staining in the ischemic rat cortical areas showed that increased numbers of PACAP38^+^ cells coexpressing with HIF-1α as well as HIF-1α nuclear translocation were inhibited by 2-ME2 injection (Fig. 2B). Upregulation of PACAP38 immunoreactivity after cerebral ischemia was also suppressed by 2-ME2 injection in a dose-dependent manner (Fig. 2C). Western blotting also demonstrated a dose-dependent 2-ME2 inhibition of PACAP38 expression after cerebral ischemia (Fig. 2D). Specially, ischemia-induced PACAP38 upregulation was not present in the brain of HIF-1α KO mice (Fig. 2E). In addition, HIF-1α expression in PCCs was localized in both the cytosol (including neurites) and nucleus under normoxia (Fig. 2F). HIF-1α translocation into the nucleus was noted under hypoxia (Fig. 2F). However, pretreatment of PCCs with 2-ME2 for 16 hours abolished HIF-1α translocation into the nucleus (Fig. 2F). PACAP38 expression was also suppressed when PCCs were pretreated for 16 hours with 2-ME2 (Fig. 2F, 2G) or HIF-1α knockdown in PCCs via lentiviral delivery of HIF-1α shRNA (LV-HIF-1α-sh) (Fig. 2G). The 2-ME2 effect was specific to HIF-1α-induced PACAP38 expression, as it did not affect lentiviral (LV-PACAP38) infection-induced PACAP38 expression (Fig. 2G).

To prove whether the hypoxia-induced PACAP38 upregulation is only through HIF-1α, overexpression of HIF-1α or HIF-2α and knockdown of HIF-1α or HIF-2α were used to address this issue. Our results demonstrated that genetic manipulation of HIF-1α via lentiviral transduction (LV-HIF-1α or LV-HIF-1α-sh) modulated the expression of PACAP38 in PCCs at 1 hour after hypoxia compared to genetic manipulation of HIF-2α (Fig. 2H). Then, ChIP assay was conducted to demonstrate interaction between HIF-1α and the PACAP promoter. HIF-1α was recruited to bind on the PACAP promoter in PCCs subjected to 4-hour hypoxia, but not in PCCs under normoxia or PCCs treated with LV-HIF-1α-shRNA under 4-hour hypoxia (Fig. 2I). Furthermore, the activity of a luciferase reporter gene construct (pPACAP-luc1) containing HRE from the PACAP gene promoter coupled to an SV40 promoter under hypoxia was much higher than a control construct (pPACAP-luc2) and an HRE-mutant construct (pPACAP-mutHRE; Fig. 2J). Taken together, these results provide direct evidence suggesting hypoxia-induced PACAP38 is through HIF-1α-dependent regulation via the direct binding to PSCAP38 promoter.

### PACAP38/PAC1 Signaling Promoted BMDC Proliferation and Trafficking

**Figure 3:**
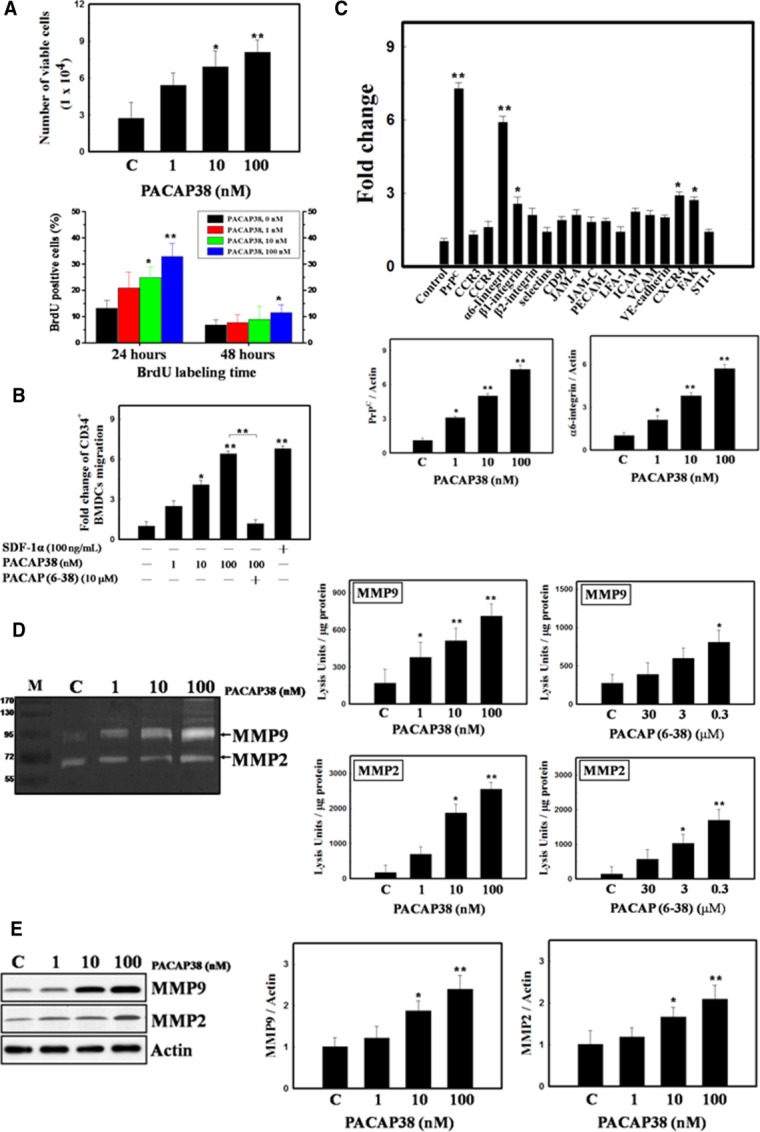
Administration of PACAP38 enhanced the proliferation and migration of BMDCs via PAC1 signaling. (A): PACAP38 significantly increased cell viability and proliferation as seen with trypan blue exclusion and BrdU labeling in a dose-dependent manner. (B): A transwell migration assay for CD34^+^ BMDCs treated with PACAP38 (SDF-1α was used as a positive control) in the absence and presence of the PAC1 antagonist PACAP (6–38). (C): Bar graph showing changes in target proteins corresponding to cell adhesion and migration in PACAP38-treated BMDCs. (D): PACAP38 activated both MMP9 and MMP2 in cell culture supernatants in a dose-dependent manner as seen with gel zymography. PACAP38 (100 nM)-induced MMP2 and MMP9 activities were abolished by administration of PACAP (6-38). (E): Western blotting showed that PACAP38 upregulated expression of MMP9 and MMP2. *n* = 8 per group. The mean ± SEM is shown. *, *p* < .05 and **, *p* < .01 vs. control. Abbreviations: BrdU, bromodeoxyuridine; BMDC, bone marrow-derived cell; CCR3, CC chemokine receptor 3; CCR4, CC chemokine receptor 4; CXCR4, CXC receptor type 4; FAK, focal adhesion kinase; ICAM, intercellular adhesion molecule; JAM-A, junctional adhesion molecular A; JAM-C, junctional adhesion molecular C; LFA-1, lymphocyte function-associated antigen 1; MMP, matrix metalloproteinase; PECAM, platelet/endothelial cell adhesion molecule 1; PACAP38, pituitary adenylate cyclase-activating peptide 38; PrP^C^, cellular prion protein; STI-1, stress-induced-phosphoprotein 1; VCAM, vascular cell adhesion molecule.

Next, we investigated the role of PACAP38 in cell proliferation and migration in vitro, we measured cell numbers and BrdU incorporation and tested the ability of CD34^+^ BMDCs to migrate in response to increasing PACAP38 concentrations. Cell proliferation based on trypan blue exclusion by viable cells and the BrdU labeling index was increased following PACAP38 administration in a dose-dependent manner (Fig. 3A). The migration of CD34^+^ BMDCs treated with PACAP38 occurred in a concentration-dependent manner (Fig. 3B). PACAP (6–38), a PAC1 antagonist, inhibited PACAP38-induced BMDC migration (Fig. 3B). To explore molecular mechanism of PACAP38 induced-cell migration and adhesion in vitro, we analyzed the protein expression of known target proteins that are involved in cell adhesion and migration in PACAP38-treated BMDCs: cellular prion protein (PrP^C^), CCR3, CCR4, α6-integrin, β1-integrin, β2-integrin, p-selectin, CD99, JAM-A, JAM-C, PECAM-1, LFA-1, ICAM, VCAM, VE-cadherin, CXCR4, FAK, and STI-1 [51–57]. Ten to twelve hours after treatment, PACAP38 upregulated the expression of the adhesion molecules PrP^C^ and α6-integrin [58,59] in a dose-dependent manner (Fig. 3C). In addition, PACAP38 also enhanced the expression and activity of matrix MMP 2 and 9 in a dose-dependent manner (Fig. 3D, 3E). PACAP (6–38) inhibited the PACAP38-induced enhancement of MMP2 and MMP9 activities (Fig. 3D), indicating PACAP38/PAC1 signaling involved in activation of MMP2 and MMP9.

### BMDCs Homes to the Vascular Niche of Ischemic Brain via Response to PACAP38/PAC1 Signaling

**Figure 4:**
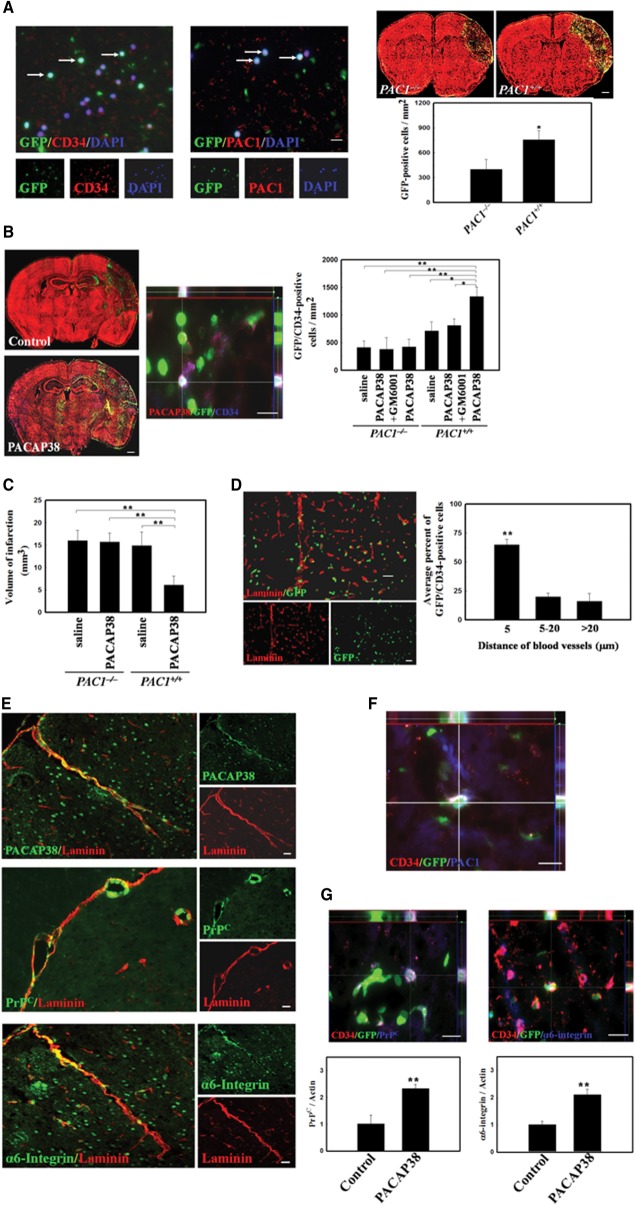
PACAP38-to-PAC1 signaling induced bone marrow-derived cells (BMDCs) homing to the vascular niche of ischemic brain. (A): Double immunofluorescence for CD34 and PAC1 in BMDCs from a peripheral blood smear from GFP-chimeric mice (left panel). Representative reconstructed brain images of GFP-chimeric mice (*PAC1^+/^^+^* or *PAC1^−/−^* mice; green color, GFP; red color, propidium iodide) 3 days after cerebral ischemia. Quantitative data from GFP^+^ cells are shown (right panel). (B): Representative reconstructed brain images with or without PACAP38 treatment labeled as in (a) 7 days postcerebral ischemia (right panel). By 3D colocalization, CD34^+^GFP^+^ were costained with PACAP38 (middle panel). Quantitative data from CD34^+^GFP^+^ cells are shown. In addition, BMDC homing was assessed in the absence and presence of the matrix metalloproteinase inhibitor GM6001 (saline, control). (C): Quantitative data from the infarct volume in PACAP38-treated *PAC1^+/^^+^* mice, saline-treated *PAC1^−/−^* mice, saline-treated *PAC1^+/^^+^* mice, and PACAP38-treated *PAC1^−/−^* mice. (D): Representative image of costaining of GFP^+^ cells (green) and laminin^+^ vasculature (red) in the penumbric region of a GFP-chimeric mouse brain 3 days after stroke. Quantification of the distance between GFP^+^ cells and the nearest laminin^+^ blood vessel surface was performed. (E): Representative immunohistochemical colocalization for PACAP38, PrP^C^, α6-integrin, with laminin in wild-type mice brain. (F): In 3D image, PAC1 coexpressed in GFP^+^CD34^+^ BMDCs. (G): Immunohistochemical costaining of GFP^+^CD34^+^ BMDCs for PrP^C^ and α6-integrin expression, and quantification of expression from Western blots for those two markers in stroke mouse brains after 3 days of PACAP38 or vehicle-control treatment. *n* = 8 per group. The mean ± SEM is shown. *, *p* < .05 and **, *p* < .01 vs. control. Scale bar = 50 μm. Abbreviations: GFP, green fluorescent protein; G-CSF, granulocyte colony stimulating factor; PACAP38, pituitary adenylate cyclase-activating peptide 38; PAC1, PACAP type 1 receptor; PrP^C^, cellular prion protein.

Based on the links of brain ischemia-induced PACAP38 expression and PACAP38/PAC1 signaling in CD34^+^ BMDCs trafficking, we hypothesized PACAP38/PAC1 signaling is critical pathway involved in brain ischemia-mediated recruitment of BMDCs to the vascular niche. To test this model, we first determine the association between CD34^+^ BMDCs homing and PACAP38/PAC1 signaling in vivo. In a double immunofluorescence study, CD34^+^ BMDCs from GFP-chimeric mice were showed colocalization of PAC1 (Fig. 4A). Moreover, significantly increased numbers of GFP^+^ BMDCs were found in the ischemic brains of *PAC1^+/^^+^* GFP-chimeric mice as compared with *PAC1^−/−^* mice on 3 days after cerebral ischemia (Fig. 4A). To determine whether BMDCs homed to the ischemic brain of PACAP38-treated mice, double immunohistochemical staining was conducted on brain sections on 7 days after cerebral ischemia in GFP-chimeric mice (*PAC1^+/^^+^* and *PAC1^−/−^* mice). A significantly increased number of GFP^+^CD34^+^ BMDCs were found in the right striatum, hippocampus, and penumbral area in PACAP38-treated *PAC1^+/^^+^* mice as compared with PACAP38-treated *PAC1^−/−^* mice, saline-treated *PAC1^+/^^+^* mice, and saline-treated *PAC1^−/−^* mice (Fig. 4B). However, PACAP38-induced BMDC homing was abolished by administration of an MMP inhibitor (GM6001) in PACAP38-treated *PAC1^+/^^+^* mice (Fig. 4B). Furthermore, the infarct volume in PACAP38-treated *PAC1^+/^^+^* mice was significantly reduced as compared with that in saline-treated *PAC1^+/^^+^* mice, PACAP38-treated *PAC1^−/−^* mice, and saline-treated *PAC1^−/−^* mice (Fig. 4C).

To establish how BMDCs integrate into the penumbra of the ischemic brain and specifically whether they home to the vasculature, we measured the relationship of BMDCs to the closest endothelial cell surface after cerebral ischemia in GFP-chimeric mice. On 3 days after stroke, the penumbric region of GFP-chimeric mouse brains was stained for laminin to visualize the blood vessels (Fig. 4D). CD34^+^GFP^+^ BMDCs were predominantly observed near the penumbric vasculature (Fig. 4D). Most CD34^+^GFP^+^ BMDCs (65% ± 4.7%) were within 5 μm of the laminin^+^ vasculature, 20% ± 3.1% were 5–20 μm away, and 16% ± 7.2% were >20 μm away. Thus, BMDCs migrate to the vasculature of the ischemic brain in vivo, consistent with their ability to home to this vascular niche.

We then examined the molecular mechanism that allowed BMDCs to be located near blood vessels. Because the PACAP is involved in adult NPC homing to areas of central nervous system (CNS) injury after ischemia [11], we tested PACAP as a candidate for recruitment of BMDCs. PACAP38 was coexpressed with the vasculature marker laminin, and expression of the migration and adhesion markers, such as PrP^C^ and α6-integrin, was seen along laminin^+^ blood vessels in the penumbric area (Fig. 4E). Importantly, we found that PAC1 was visible on GFP^+^ BMDCs associated with the vasculature after homing to the ischemic brain of GFP-chimeric mice (Fig. 4F). Thus, the ligand, PACAP38, was enriched in the niche, and GFP^+^ BMDCs express the receptor, PAC1, consistent with the hypothesis that this ligand-receptor pair is involved in BMDC homing to the stroke brain. In addition, PrP^C^ and α6-integrin were also coexpressed in CD34^+^GFP^+^ BMDCs (Fig. 4G). Consistent with the results from BMDC culture, PACAP38 administration in ischemic brain significantly increased PrP^C^ and α6-integrin expression (Fig. 4G). Taken together, PACAP38 treatment alters the expression of key receptors associated with proliferation, adhesion, and migration of BMDCs, providing pleiotropic effects on the BMDC lineage, similar to its effects on the NPC lineage [11].

### PACAP38/PAC1 Signaling Facilitated Stroke Recovery by BMDC Trafficking into Brain

**Figure 5:**
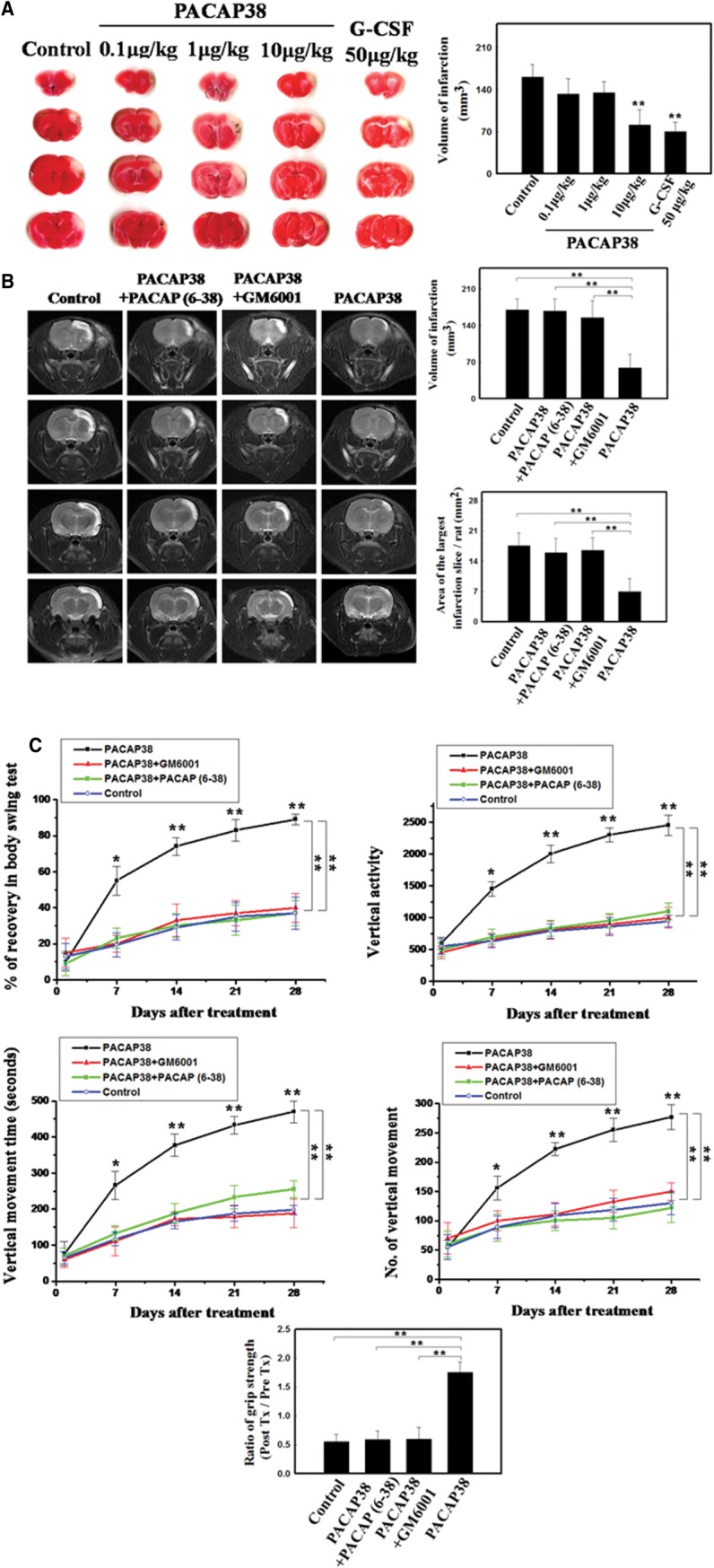
Promotion of stroke recovery by bone marrow-derived cells recruitment via response to PACAP38-to-PAC1 signaling. (A): Representative triphenyltetrazolium chloride staining (white area indicates the infarction) of rat brain sections (four sections per one rat) 3 days after cerebral ischemia (50 μg/kg granulocyte colony-stimulating factor was used a positive control). Quantitative data from infarction volume are shown. (B): Magnetic resonance imaging of rat brains 7 days after cerebral infarction (white area) with the indicated treatments. Quantification data from infarction volume are shown for four sections per brain in each group. (C): Neurological behavior measurements including body swing tests, locomotor activity tests (vertical activity, vertical movement time, and number of vertical movements), and grip strength tests in rats after cerebral ischemia, with the indicated treatments. *n* = 8 per group. The mean ± SEM is shown. *, *p* < .05 and **, *p* < .01 vs. control. Scale bar = 50 μm. Abbreviations: G-CSF, granulocyte colony stimulating factor; PACAP38, pituitary adenylate cyclase-activating peptide 38.

Our above results suggest rPACAP38 as an ischemia-inducible endogenous factor for mobilization of BMDCs into the circulation that are subsequently recruited to the ischemic brain tissue. According to this novel mechanism, we next proposed the supplement of exogenous PACAP38 as therapeutic approach may promote the homing of BMDCs to ischemic areas and neural survival. To select the most effective treatment dosage of PACAP38, TTC staining was measured in three rat groups treated with 0.1, 1, or 10 μg/kg PACAP38. The infarct volume of the rats given 10 μg/kg PACAP38 was much smaller than that in the other dosage groups at 3 days after cerebral ischemia (Fig. 5A). At 7 days after cerebral ischemia, the infarct volume and area of the largest infarcted slice as assessed by MRI were significantly reduced in PACAP38-treated mice as compared with those in the groups treated with PACAP38 + GM6001, PACAP38 + PACAP (6–38), or saline (Fig. 5B). Body asymmetry, locomotor activity tests, and grip strength measurement were used to assess neurological deficit recovery in PACAP38-treated, PACAP38+GM6001-treated, PACAP38+PACAP (6–38)-treated, and control rats. PACAP38-treated rats showed better recovery in body swing tests than did rats treated with PACAP38 + GM6001, PACAP38 + PACAP (6–38), or saline control (Fig. 5C). Locomotor activities were substantially better after cerebral ischemia in rats receiving PACAP38 as compared with the other groups (Fig. 5C). In addition, comparison of forelimb grip strength before and 28 days after ischemia showed that the PACAP38-treated group had a much better grip strength ratio than did the other groups (Fig. 5C).

### Promotion of Neural Survival and BMDC-Related Angiogensis by PACAP38/PAC1 Signaling

**Figure 6:**
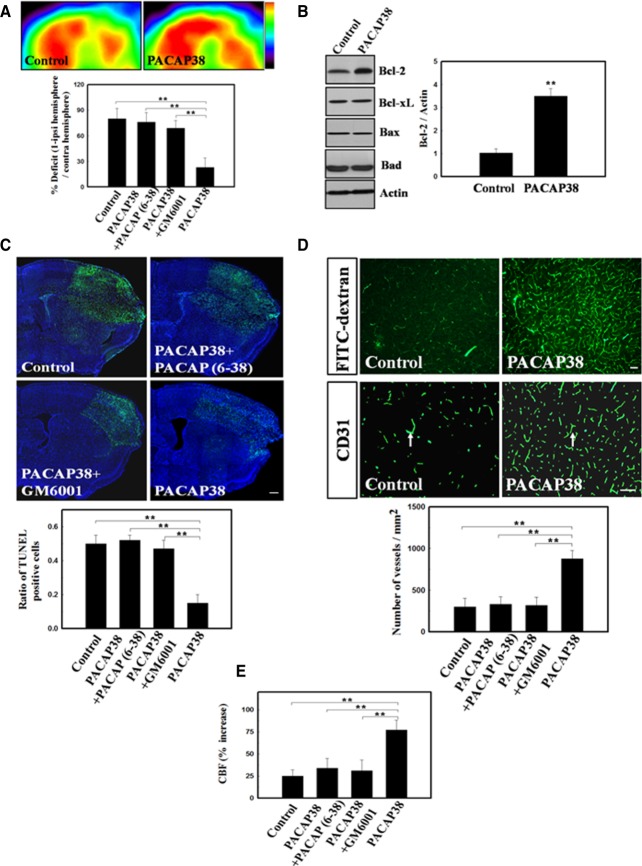
PACAP38-to-PAC1 (PACAP type 1 receptor) signaling enhanced neural survival and bone marrow-derived cell-related angiogensis. (A): Representative microPET images, semiquantitative measurement of relative glucose metabolic activity based on FDG uptake are shown. (B): Western blotting for Bcl-2, Bcl-xL, Bax, and Bad in PACAP38-treated and control rats 3 days after cerebral ischemia. Quantitative data from Western blot are shown. (C): Representative images of the TUNEL assay (green) and Hoechst 33342 (blue) costaining. Semiquantitative data for TUNEL^+^ cells are presented. (D): FITC-dextran-perfused brain samples indicate the microvascular pattern in PACAP38-treated and control rats. Cerebral blood vessel density was quantified with CD31 immunoreactivity (white arrows). (E): Semiquantitative measurement for laser doppler flowmeter monitoring of CBF in the ischemic cortex of rats treated with PACAP38, PACAP38 + GM6001, and PACAP38 + PACAP (6–38) and of control rats. *n* = 8 per group. The mean ± SEM is shown. *, *p* < .05 and **, *p* < .01 vs. control. Scale bar = 50 μm. Abbreviations: CBF, cerebral blood flow; FITC, fluorescein isothiocyanate; PACAP38, pituitary adenylate cyclase-activating peptide 38; TUNEL, terminal deoxynucleotidyl transferase-mediated digoxigenin-dUTP nick-end labeling.

To verify whether PACAP38 enhanced metabolic activity, cortical glucose metabolism was examined with ^18^F-FDG PET imaging at 1 week after treatment. The microPET images of the right cortex (the site of cerebral ischemia) of the PACAP38-treated group showed a significant increase in FDG uptake, which was higher than that in the other three groups **(**Fig. 6A). The antiapoptotic effects of PACAP38 in the ischemic brain were examined with Western blotting of apoptosis-related proteins. We observed significantly upregulated expression of the antiapoptotic protein Bcl-2 in PACAP38-treated rats 24 hours after cerebral ischemia as compared with that in control rats (Fig. 6B). Cellular apoptosis in ischemic rat brain was studied with TUNEL staining. The penumbral region surrounding the ischemic core of PACAP38-treated rats contained fewer TUNEL**^+^** cells than that of the other three groups (Fig. 6C).

To determine whether intraperitoneal PACAP38 administration induced angiogenesis by promoting the homing of BMDCs to ischemic sites FITC-dextran perfusion studies and blood vessel density assays were performed on each brain slice from each experimental rat. Visual inspection of FITC-dextran perfusion indicated that PACAP38 induced much more cerebral microvascular perfusion than did saline (Fig. 6D). Blood vessel density assays showed that ischemic rats treated with PACAP38 had better neovascularization in the penumbral area than did the other groups (Fig. 6D). We next used LDF to examine whether the increased blood vessel density enhanced functional CBF in the ischemic brains. One week after cerebral ischemia, CBF in the ischemic cortex of the PACAP38-treated rats was significantly increased as compared with that of the other three groups (Fig. 6E).

## Discussion

Demonstrating whether stem cells home to vascular cells is an important issue because this interaction could allow BMDCs to integrate into vascularized parenchymal regions of the ischemic brain to enhance tissue repair. Here we show that BMDCs homed toward endothelial cells under ischemic stress and provide the direct evidence for supporting PACAP38/PAC1 axis-mediated signals involved in these processes. The rationale of endothelial cells expressing high levels of PACAP38, which are required for NPC homing to the ischemic brain, let us explore the role of PACAP38 in BMDCs homing to vascular niches [11]. Double immunofluorescence staining in ischemic stroke rat brains showed an expression pattern consistent with PACAP38 being synthesized by blood vessels and released into the nearby environment. We found that the PACAP38 receptor, PAC1, is widely expressed on BMDCs. Although PACAP38 may contribute to the build-up of neural lineage cells around blood vessels in the long term, it is unlikely that cells preferentially survive or proliferate near blood vessels. Pursuing the hypothesis that homing was responsible, we demonstrated that blood vessel endothelial cells secrete factors of PACAP38 that elicit BMDC chemotaxis when provided as a gradient. Furthermore, PACAP38 upregulates α6-integrin and PrP^C^ expression on the surface of BMDCs [60,61]. This may stimulate BMDCs to move toward blood vessels and increase their binding to laminin, which is concentrated on blood vessel surfaces. Importantly, homing of BMDCs to the penumbric area of ischemic brain was effectively blocked in PACAP (6–38)-treated and in *PAC1^−/−^* mice, demonstrating that PACAP38-PAC1 signaling is a critical component of the homing mechanism. The observation that PACAP38 level was important for BMDC homing to endothelial cells provides a satisfying parallel with the homing mechanism of NPCs [12,13]; pursuing other molecular parallels in these pathways will be important.

PACAP is a potent stimulator of cAMP accumulation and gene expression [15,62]. PACAP participates in immunomodulation [63] and is related to the etiology of neuropsychiatric disease [64]. Although some investigators have discovered PACAP upregulation following cerebral ischemia in animals [6] and neural cell culture [65], we also demonstrated that increased PACAP38 expression was induced in human and rat ischemic brain. This is consistent with high PACAP expression under stress conditions [66]. Thus, PACAP may be expressed in response to stress [67]. In agreement with previous studies, PACAP seems to have similar functions as some trophic factors, such as the activation of endogenous protective mechanisms to enhance growth and repair [68] of injured neural tissue. In the cerebral ischemia and hypoxia model especially, PACAP38 activation may transduce an important signal for neural adaptation to ischemia-related environmental stress.

Although Stumm et al. [6] have found that cerebral ischemia induces *N*-methyl d-aspartate (NMDA) receptor-mediated upregulation of PACAP, the cellular and molecular mechanisms of the PACAP upregulation are unclear. The novel contribution of our study is that hypoxia/ischemia-induced expression of PACAP38 was mediated by the activation and binding of HIF-1α to the HRE of the PACAP38 promoter. The level of HIF-1β did not change during the hypoxia/ischemia. Hypoxia/ischemia-induced PACAP38 upregulation was abolished in neural tissues with decreased HIF-1α activity that resulted from the injection of the HIF-1α-specific inhibitor, 2-ME2; in animals with cerebral ischemia and in a Cre-loxP-based approach to knockout HIF-1α in mice. The proximal promoter region (∼0.8 kb) of the mouse PACAP gene has at least one putative HIF-1α binding site with the consensus sequence 5′-ACGTG-3′ [69]. In a reporter assay, hypoxia-induced luciferase activation was abolished after mutation of this potential HIF-1α binding site. Moreover, binding of HIF-1α to this putative HRE was demonstrated by ChIP assays in hypoxic neurons in culture.

The mobilization and homing of BMDCs are enhanced by factors such as granulocyte colony-stimulating factor (G-CSF), vascular endothelial growth factor (VEGF), SDF1-α and stem cell factor, as well as appear to involve a receptor-mediated process that includes the G-CSF receptor, VEGF receptor, CXCR4, and c-kit [7,70–75], respectively. Although some investigators have focused on PACAP-mediated proliferation and migration of NPCs [11,76], few reports have studied the relationship between BMDC homing and PACAP expression [15]. In previous studies, activation of cAMP and downstream signaling by PACAP not only enhance CD34^+^ cell survival and homing but also mediate G-CSF-induced CD34^+^ cytoprotection [77]. In addition, cAMP activation upregulates CXCR4 expression to augment the motility of CD34^+^ cells [78]. Here we demonstrated that endogenous PACAP38 secretion in the ischemic brain promoted BMDC mobilization, which appeared to be a PAC1-associated process. Regarding the molecular mechanism of BMDC trafficking, PACAP38 increase the expression of adhesion/migration related proteins including PrP^C^, α6-integrin, β1-integrin, FAK, and CXCR4 [51–57], as well as enhance the activity of MMP9 and MMP2 in BMDCs to promote homing and migration. Therefore, we assume that PACAP38 induces PAC1-mediated signaling as a major pathway that regulates BMDC trafficking.

We found that the angiogenic effect played an important role in the neuroprotective outcome in the ischemic brain. Administration of PACAP38 seemed to augment angiogenesis in the penumbral area after stroke. Although some investigators have demonstrated that the angiogenic action of PACAP is mediated by VEGF stimulation [4,79], few reports have focused on direct angiogenesis by PACAP. Since one important function of HIF-1α is to promote tissue angiogenesis, HIF-1α can guide migration of endothelial precursor cells toward a hypoxic environment [80]. Here we discovered that PACAP38 upregulation by hypoxia/ischemia via HIF-1α activation stimulated neovascularization, thus increasing CBF in the penumbral area. Therefore, PACAP38 may be considered a new angiogenic factor that is regulated by HIF-1α induction.

## Conclusions

We present experimental findings to support the contention that PACAP38 expression was upregulated by hypoxia/ischemia via activation of HIF-1α. To the best of our knowledge, HIF-1α transactivation of PACAP38 has not been previously reported. We further demonstrate that the activation of the PACAP38-PAC1 signaling cascade is pivotal for facilitating BMDC homing toward the ischemic niche in the brain to confer neuroprotective as well as angiogenic actions in reducing brain damage and in enhancing functional recovery after cerebral ischemia. The clinical significance of the PACAP38-PAC1 cascade on BMDC homing is further strengthened by the findings that exogenous PACAP38 is capable of exerting the same action as the endogenous counterpart to provide a possibly novel therapeutic intervention that may improve regeneration and repair following cerebral ischemia.
